# Supplementary material for: Chromothripsis during telomere crisis is independent of NHEJ, and consistent with a replicative origin
Source: Genome Res. 2019 May;29(5):737–49. doi: 10.1101/gr.240705.118 (PMC6499312; doi:10.1101/gr.240705.118)
Supplement: Supplemental Material [file supp_gr.240705.118_Supplemental_file_1.zip › contigs/annotated_contigs/DB80/contig.2.DB80_length_400_mean_cov_7.86.docx]

**DB80_length_400_mean_cov_7.86**

```
AGTGGGAAGTGAAAGGGTTGTGGTAAGCATGGGCTGTGCGTGGAGCTGGCGTGCTACCCTCTGCATCATCCATGGAAATGCCCGACTCA
 >chr3:17788692-17788809 + E=6e-59
GATCTCCTGCTGCAAGAACATAGTTGAC|TTACATTTTGTAAGTCCC|ATTTTTGGTATTTTTAGTAGAGACGGGGTTTCACCATGTTG
                                               >chr3:17790043-17790308 + E=4e-148
GCCAGGATGGTCTCAATCTCTTGACCTCATGATCCACCCACCTCAGCCTTCCAAACTGCTGGGATTACAGGCATGAGCCACCGCACCCG

GCCATACCATTCTGTGTTTCTGGAGCAGATACTTTCAGAGTTATTTGTCTAGCTCATAACAAGAACCATCATCCCAGCACTTTGGGAAG

CCGAGGCGGGCGGCTCACCTGAGGTCCGGAGTTCGAGACCAGCCTG
```
